# Pattern of Mitochondrial Respiration in Peripheral Blood Cells of Patients with Parkinson’s Disease

**DOI:** 10.3390/ijms231810863

**Published:** 2022-09-17

**Authors:** Tommaso Schirinzi, Illari Salvatori, Henri Zenuni, Piergiorgio Grillo, Cristiana Valle, Giuseppina Martella, Nicola Biagio Mercuri, Alberto Ferri

**Affiliations:** 1 Unit of Neurology, Department of Systems Medicine, Tor Vergata University of Rome, 00133 Rome, Italy; 2 IRCCS Fondazione Santa Lucia, 00179 Rome, Italy; 3 National Research Council, Institute of Translational Pharmacology (IFT), 00179 Rome, Italy

**Keywords:** Parkinson’s disease, PBMCs, mitochondria, Seahorse, immunometabolic pathway, neuroinflammation, biomarkers, synaptopathy

## Abstract

Mitochondria are central in the pathogenesis of Parkinson’s disease (PD), as they are involved in oxidative stress, synaptopathy, and other immunometabolic pathways. Accordingly, they are emerging as a potential neuroprotection target, although further human-based evidence is needed for therapeutic advancements. This study aims to shape the pattern of mitochondrial respiration in the blood leukocytes of PD patients in relation to both clinical features and the profile of cerebrospinal fluid (CSF) biomarkers of neurodegeneration. Mitochondrial respirometry on the peripheral blood mononucleate cells (PBMCs) of 16 PD patients and 14 controls was conducted using Seahorse Bioscience technology. Bioenergetic parameters were correlated either with standard clinical scores for motor and non-motor disturbances or with CSF levels of α-synuclein, amyloid-β peptides, and tau proteins. In PD, PBMC mitochondrial basal respiration was normal; maximal and spare respiratory capacities were both increased; and ATP production was higher, although not significantly. Maximal and spare respiratory capacity was directly correlated with disease duration, MDS-UPDRS part III and Hoehn and Yahr motor scores; spare respiratory capacity was correlated with the CSF amyloid-β-42 to amyloid-β-42/40 ratio. We provided preliminary evidence showing that mitochondrial respiratory activity increases in the PBMCs of PD patients, probably following the compensatory adaptations to disease progression, in contrast to the bases of the neuropathological substrate.

## 1. Introduction

Parkinson’s disease (PD) is a common neurodegenerative disorder, responsible for both motor and non-motor disturbances, whose neuropathological hallmarks are the loss of dopaminergic nigral cells and the brain accumulation of α-synuclein (α-syn)-positive Lewy bodies [1,2].

Mitochondria are highly plastic and dynamic organelles, and are critical for cellular bioenergetics and neuronal homeostasis [3]. Mitochondrial dysfunction and PD pathogenesis have been historically connected. Solid evidence from genetics and experimental models [4,5] shows how dysfunctional mitochondria lead to oxidative stress and synaptopathy, namely the earliest neurodegeneration steps in PD [6,7]. Mitochondrial impairment also precipitates lysosomes activity, protein turn-over, and α-syn metabolism, triggering or fostering neurodegeneration at every stage of PD [8]. Moreover, mitochondria are central in neuroinflammation and immune response [9], other critical determinants of PD pathology [10,11]. Accordingly, mitochondria-related immunometabolic pathways could emerge as alternative targets for disease-modifying treatments in PD [12], useful for the substitution or integration of anti-α-syn drugs, which failed to halt disease progression alone [8,13,14].

From this perspective, the role of mitochondria in PD must be further elucidated. In particular, we have to overtake current knowledge, mostly relying on preclinical studies [15], and acquire evidence directly from patients.

Peripheral blood mononucleate cells (PBMCs) exhibit typical PD-neuropathology signatures [1,16]. In addition, immune cells directly participate in the pathogenic cascade of PD, either at the central or peripheral level [11], thus representing the circulating leukocytes as an ideal tissue to analyze in vivo molecular events underlying PD.

In this study, we shaped the pattern of mitochondrial bioenergetics in vivo in PD. We thus assessed mitochondrial respiration in the PBMCs of PD patients and examined the respective correlations with clinical features and levels of cerebrospinal fluid (CSF) neurodegeneration biomarkers (α-synuclein, amyloid-β peptides, tau proteins and lactate), which are considered as an indication of pathological changes in the brain [17].

Indeed, mitochondrial respiration is a key function in cellular homeostasis, an epicenter of many metabolic pathways that are crucial for the clinical–pathological progression of PD [18], whose deeper knowledge is fundamental to design novel therapeutic intervention strategies in PD.

## 2. Results

### 2.1. Study Population

Table 1 summarizes the clinical and demographic data of the study population. Groups were homogeneous regarding age and sex distribution.

### 2.2. Bioenergetic Parameters

**Figure 1:**
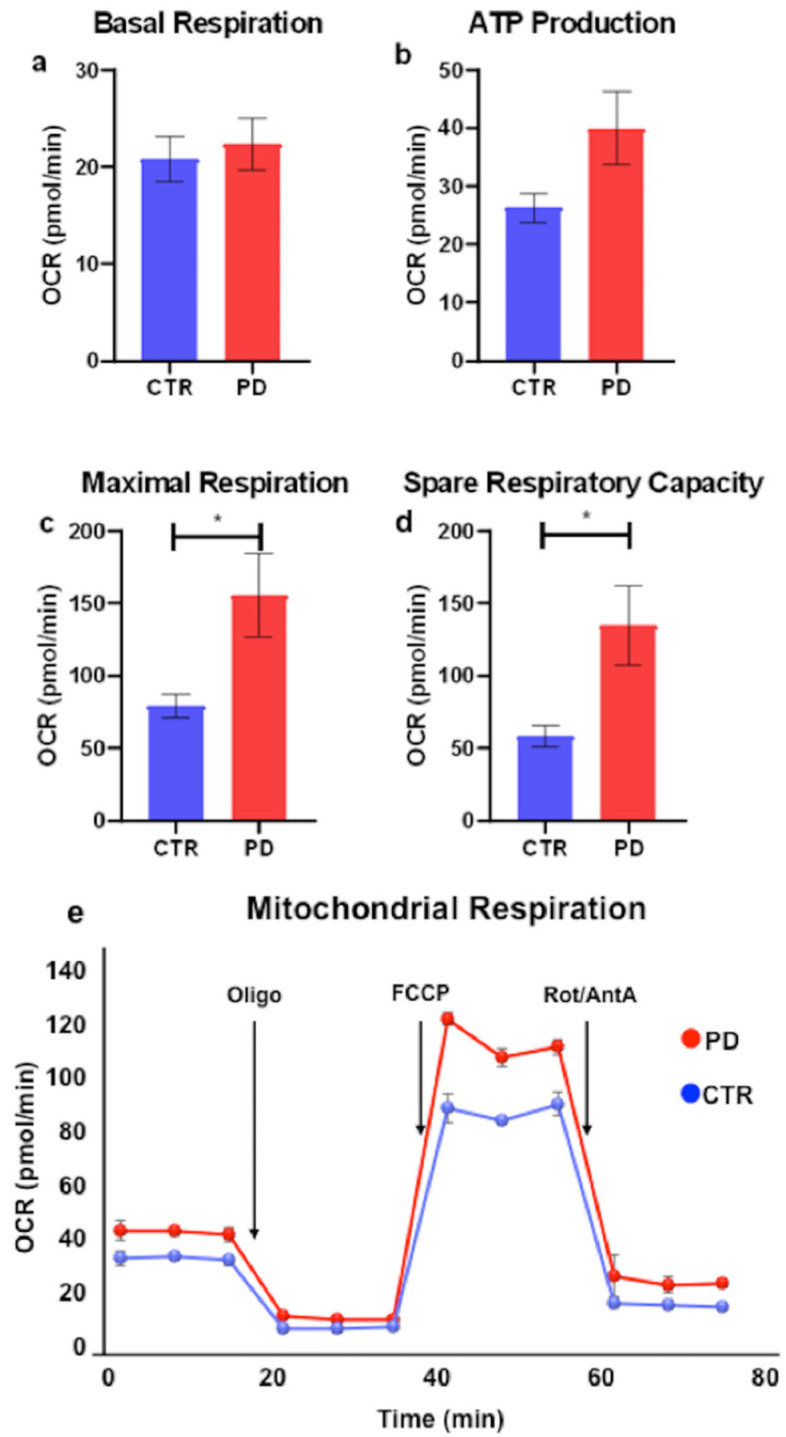
**PBMC respirometry by Seahorse Bioscience.** (**a**–**d**) Bar graphs showing differences in bioenergetic parameters between PD and controls (values are expressed as means ± S.E.M; * = *p* < 0.05). (**e**) Representative time course of OCR during an experimental session (one patient and one control).

PBMCs obtained from PD patients and controls had a similar baseline oxygen consumption rate (OCR) (mean ± st.dev. in pmol/min) (PD = 22.4 ± 10.6; controls = 20.8 ± 8.8). ATP-linked respiration was higher in PD (40.2 ± 23.4) than in the controls (26.3 ± 9.4), although it was not statistically significant (*p* = 0.07). Maximal respiration was significantly higher in PD (155.6 ± 115.0) than in the controls (79.3 ± 29.9, *p* = 0.038). The spare (or reserve) respiratory capacity was significantly higher in PD (134.8 ± 108.2) than in the controls (58.5 ± 28.04, *p* = 0.02) (Figure 1a–e). There were no significant gender differences in either of the groups. Sample normalization was optimized through Western Blot analysis (Supplementary Figure S1).

### 2.3. Correlation Analysis

Maximal respiration was directly correlated with disease duration (R = 0.5, *p* = 0.04), Hoehn and Yahr scale (H & Y) stage (R = 0.7, *p* = 0.003), and MDS-UPDRS part III (R = 0.62, *p* = 0.01). Spare respiratory capacity was directly correlated with disease duration (R = 0.5, *p* = 0.04), H & Y stage (R = 0.7, *p* = 0.004), and MDS-UPDRS part III (R = 0.63, *p* = 0.009). No further correlations emerged between bioenergetic parameters and clinical features (age, Mini-Mental State Examination (MMSE), Montreal Cognitive Assessment (MOCA) and Non-Motor Symptoms Scale (NMSS) scores, and levodopa equivalent daily dose (LEDD)).

Spare respiratory capacity was directly correlated with both amyloid-β-42 (Aβ42) and amyloid-β-42/amyloid-β-40 (Aβ42/Aβ40) ratio (R = 0.68, *p* = 0.02 and R = 0.66, *p* = 0.04, respectively). No further correlations between bioenergetic parameters and other CSF biomarkers (α-syn, total-tau (t-tau) and phosphorylated-tau (p-tau), lactate, and the CSF/blood albumin ratio) were found.

## 3. Discussion

PBMCs offer the opportunity to track the in vivo molecular events underlying PD, serving as a reliable model for central neuropathology [1,16]. Here, we shaped the pattern of mitochondrial respiration in PD by applying Seahorse Bioscience technology to the PBMCs of both PD patients and healthy controls, and examining the correlations of bioenergetic parameters with clinical features and levels of CSF biomarkers of neurodegeneration.

We found that, in PD, PBMCs had a peculiar pattern of mitochondrial respiration, with normal basal respiration, a significant increase in both maximal respiratory capacity and spare respiratory capacity, and a higher (but not statistically significant) ATP production. The increase in maximal respiratory capacity and spare respiratory capacity was directly associated with disease duration and severity of motor impairment, suggesting that mitochondrial respiration capability could rise in parallel with the clinical–pathological progression of PD. In addition, the spare respiratory capacity was greater in patients with higher CSF levels of Aβ42 and Aβ42/Aβ40 ratio, which identifies individuals with a lower burden of cerebral amyloidopathy [17].

In contrast with theoretical expectances, which would have predicted a reduction in bioenergetic activity in the mitochondria of PD patients [8,15,19], we discovered an increase in respiratory capacity. Albeit surprising, these data are consistent with previous findings from other experimental settings. In fact, lymphoblasts of patients with PD, namely immortalized cells derived from peripheral blood lymphocytes, also showed a dramatic increase in mitochondrial respiration, ATP synthesis and maximum OCR. The greater energy production found by Annesley et al. was assumed to satisfy an increased requirement due to the higher mitochondrial turn-over induced by pathological α-syn accumulation, or, in general, to an abnormal metabolic state with high energy consumption [20]. However, the changes in respiratory activity could follow modifications in the lipid composition of different cell compartments or in lipid trafficking overall, which markedly affect the mitochondrial architecture, the transport of proteins into mitochondria, and the functioning of respiratory proteins [21]. Indeed, mitochondrial membrane potential could be altered in the PBMCs of PD patients, suggesting a certain degree of stress in those organelles [22]. Alternatively, the growing neuroinflammatory state or the greater immune activation against progressive neurodegeneration might account for the increased bioenergetics of leukocytes in PD [20]. In addition to PBMCs, fibroblasts from PD patients also displayed higher mitochondrial respiratory rates compared to healthy controls. This has been observed either in Parkin mutant carriers [23] or in subjects with the idiopathic form [24], and interpreted as a compensatory bioenergetics activation or as a consequence of abnormal mitochondrial functioning.

In line with the hypothesis that the implementation of PBMC bioenergetic activity could help cellular compensations to the progression of neuropathology and neuroinflammation, we noticed that maximal and spare respiratory capacities tended to increase with disease evolution, either in terms of disease duration or clinical impairment.

Nuclear factor erythroid 2-related factor 2 (Nrf2) is a transcription factor regulating the cellular defense against oxidative stress, inflammation and neurodegeneration, by promoting overall mitochondrial respiration and metabolism [25]. We recently demonstrated that Nrf2 was highly expressed and its pathway activated in the PBMCs of PD patients, especially in those with a longer disease duration [1]. It is thus reasonable that the progressive increase in respiratory capacity over the clinical–pathological course of PD may follow an Nrf2-mediated mitochondrial activation aimed at sustaining a systemic defensive response. Of interest, similar findings were observed in the lymphoblasts of patients with Amyotrophic Lateral Sclerosis, where the respiratory activity was increased together with the upregulation of the Nrf2 pathway, suggesting this axis as a common compensation mechanism among different sporadic neurodegenerative diseases [26].

Then, we found that, in PD patients, the respiratory capacity was directly correlated with CSF levels of the Aβ42 to Aβ42/Aβ40 ratio. Experimental models of Alzheimer’s disease showed that neurons exhibit mitochondrial dysfunction and respiration impairment in the presence of Aβ42 peptides [27]. A reduction in CSF Aβ42 corresponds to the greater brain accumulation of amyloid-β plaques even in PD [17]. Accordingly, we could hypothesize that the poorer respiratory activity in patients with lower CSF Aβ42 (and worse amyloidopathy in the CNS) reflects a detrimental association between mitochondrial performances and pathological amyloid peptides, as well as at a peripheral level. In fact, PD patients with lower CSF Aβ42 present a more malignant and frailer phenotype [28], which is consistent with the weakening of defensive or compensatory mechanisms.

In contrast, other CSF biomarkers were not correlated with bioenergetic parameters, preventing further suppositions on the interactions between central neuropathology and systemic reactions.

Although limited by the sample size, this study demonstrated that PBMC mitochondria in PD patients had a peculiar pattern of respiration, with increased maximal and spare respiratory capacities. Respiratory changes probably reflect the increased energetic requirement due to the clinical–pathological progression of the disease and the subsequent compensatory adaptations. Such changes vary depending on the disease stage and the neuropathological substrate; they are more contained in patients with biochemical signatures of frailty.

We could interpret our results in two ways. Accepting PBMCs as a model of central neuropathology, they allow for a better understanding of the role of mitochondria in PD, profiling those dynamics that may underlie PD pathology throughout its course. On the other hand, they provide additional evidence on the presence of metabolic abnormalities in systemic immune cells, which could be useful in developing novel therapeutic strategies. Indeed, mitochondria-related metabolic pathways could be specifically targeted in immune cells to modulate their activity either at the central or systemic level, counteracting neuroinflammation and neurodegeneration. Further investigations on larger replication cohorts are now necessary to confirm and extend our findings.

## 4. Methods

### 4.1. Study Population and Biosampling

This study involved sixteen PD patients and fourteen sex/age-matched healthy controls enrolled at Tor Vergata University Hospital (Rome, Italy) in 2021–2022. PD was diagnosed according to MDS 2015 Postuma’s criteria. Controls were healthy volunteers without a history or clinical signs of neurological diseases. Subjects with main acute/chronic infectious/inflammatory/internal diseases and/or abnormal blood cell count were excluded [1].

For each subject, demographics, anthropometrics, and medical history were collected. PD patients were assessed using the Hoehn and Yahr scale (H & Y), MDS-UPDRS part III, Non-Motor Symptoms Scale (NMSS), Mini-Mental State Examination (MMSE) adjusted for age and educational level, Montreal Cognitive Assessment (MOCA), and the levodopa equivalent daily dose (LEDD) calculation.

All participants underwent venous blood sampling (20 mL), in the morning, after overnight fasting (morning drugs allowed). Blood was immediately processed to separate PBMCs through density gradient centrifugation with Ficoll-Hypaque (GE Healthcare Life Sciences), according to standard procedures. PBMCs were carefully frozen in cryoSFM medium, stored in liquid nitrogen and subsequently thawed for bioenergetics analyses [29].

Ten PD patients also underwent cerebrospinal fluid (CSF) analysis for neurodegeneration-related biomarker measurement. CSF was obtained through lumbar puncture, which was performed following standard procedures. The CSF levels of total α-synuclein (α-syn), amyloid-β-42 (Aβ42) and amyloid-β-40 (Aβ40), total-tau (t-tau) and phosphorylated-tau (p-tau), lactate, and the CSF/blood albumin ratio were quantified as previously described [2,30]. The Aβ42/Aβ40 and the Aβ42/p-tau ratios were then calculated.

The study was approved by the local EC (protocol n° 16.21), following the principles of the declaration of Helsinki. All participants signed informed consent.

### 4.2. Bioenergetics Analysis

Mitochondrial function was determined using a Seahorse XF96e Analyzer (Seahorse Bioscience—Agilent, Santa Clara, CA, USA) [31]. The PBMCs were plated at the density of 15 × 10^4^ cells/well. An equal number of cells, from each sample, was processed for a quantitative Western Blot assay (as described below). A mitochondrial stress test was performed according to Agilent’s recommendations. In brief, growth medium was replaced with XF test medium (Eagle’s modified Dulbecco’s medium, 0 mM glucose, pH = 7.4; Agilent Seahorse) supplemented with 1 mM pyruvate, 10 mM glucose and 2 mM L-glutamine. Before the assay, the PBMCs were incubated in a 37 °C incubator without CO_2_ for 45 min to allow the pre-equilibration of the assay medium. The test was performed by first measuring the baseline oxygen consumption rate (OCR), followed by sequential OCR measurements after the injection of oligomycin (1.5 µM), carbonyl cyanide 4-(trifluoromethoxy) phenylhydrazone (1 µM) (FCCP) and Rotenone (0.5 µM) + Antimycin A (0.5 µM). This allowed the measurement of the key parameters of the mitochondrial function, including the basal respiration, the ATP-linked respiration (obtained through the oligomycin-induced inhibition of ATP synthase with a subsequent decrease in electron flow through the electron transport chain, ETC), the maximal respiration (obtained through the uncoupling effect of FCCP, which induces ETC to operate at maximal capacity), the spare respiratory capacity (the difference between maximal respiration and basal respiration), and the non-mitochondrial ATP production (obtained through the rotenone/antimycin A-induced inhibition of both complex I and III).

### 4.3. Western Blot Analysis

Western Blot analysis was performed on protein extracts according to Scaricamazza et al. 2022 [32]. Specifically, 15 × 10^4^ PBMCs from each sample were lysed in 100 μL RIPA buffer (50 mM Tris–HCl pH 7.4, 0.5% Triton X-100, 0.25% Na-deoxycholate, 0.1% SDS, 250 mM NaCl, 1 mM EDTA and 5 mM MgCl_2_), 25 μL of which was loaded on SDS–polyacrylamide gel electrophoresis and transferred to nitrocellulose membranes (Perkin Elmer, Cat# NBA085B). Membranes were probed using β-Actin antibody (Santa Cruz Biotechnology Inc., Cat# sc-47778, WB 1:5000).

### 4.4. Statistical Analysis

The distribution of variables was preliminarily examined using the Shapiro–Wilk test, and the non-normally distributed variables were Log10+1 transformed for analysis when necessary. Categorical variables were compared using a chi-square test. Group analysis was conducted via parametric (one-way ANOVA or Student’s *t*-test) or non-parametric tests, as appropriate; correlations were evaluated using Spearman’s test. Statistical significance was set at *p* < 0.05. A blind analysis was run using IBM-SPSS-23. Data are available from the authors upon reasonable request.

## Figures and Tables

**Table 1:** **Demographics, clinical parameters and CSF biomarkers of the study population.** Age and disease duration are expressed in years; biomarkers in pg/mL. F = female; M = male; ns = not significant; other abbreviations are spelled out in the text.

	PD		Controls		Significance
Sex F/M (%)	6/10 (38/62%)		6/8 (42/58%)		ns
	*mean*	*st.dev.*	*mean*	*st.dev.*	
Age	66.7	7.5	64.1	11.3	ns
Disease Duration	3.25	2.38	-		-
H & Y	2.1	0.68	-		-
MDS-UPDRS part III	34.1	12.39	-		-
MMSE	27.5	3.01	-		-
MOCA	25.9	4.7	-		-
NMSS	49.9	38.6	-		-
LEDD	350.25	384.6	-		-
α-syn	761.66	287.9	-		-
Aβ42	996.1	340.16	-		-
Aβ40	6169.33	2052.29	-		-
Aβ42/Aβ40	0.16	0.046	-		-
t-tau	251.89	156.29	-		-
p-tau	42.01	29.8	-		-
Aβ42/p-tau	34.7	19.7	-		-
Lactate	1.4	0.25	-		-
Albumin Ratio	7.16	2.15	-		-

## Data Availability

Data are available from the authors upon reasonable request.

## Supplementary Materials

The following supporting information can be downloaded at: https://www.mdpi.com/article/10.3390/ijms231810863/s1
