# Supplementary material for: Pattern of Mitochondrial Respiration in Peripheral Blood Cells of Patients with Parkinson’s Disease
Source: Int J Mol Sci. 2022 Sep 17;23(18):10863. doi: 10.3390/ijms231810863 (PMC9506016; doi:10.3390/ijms231810863)

Supplementary Figure S1

Representative Western blot analysis using antibodies against -actin on protein extracts from PBMCs of a group of PD and controls.

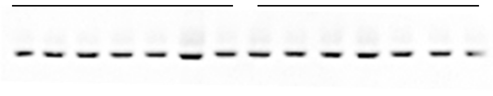

Supplement: Supplementary file 1 [file ijms-23-10863-s001.zip › ijms-1896357-supplementary.pdf]
